# Oxidative stress biomarkers in assisted reproductive technologies: From follicular redox biology to clinical translation

**DOI:** 10.1016/j.redox.2026.104167

**Published:** 2026-04-13

**Authors:** Nuan Lin, Koen van Zomeren, Torsten Plosch, Naomi Hofsink, Xiaoling Zhou, Uwe J.F. Tietge, Astrid Cantineau, Romana Schirhagl, Annemieke Hoek

**Affiliations:** a Department of Obstetrics and Gynecology, University of Groningen, University Medical Centre Groningen, Groningen, the Netherlands; b Department of Obstetrics and Gynecology, The First Affiliated Hospital of Shantou University Medical College, Shantou, China; c Center for Reproductive Medicine, Shantou University Medical College, Shantou, 515041, China; d Division of Clinical Chemistry, Department of Laboratory Medicine, Karolinska Institutet, Stockholm, Sweden; e Clinical Chemistry, Karolinska University Laboratory, Karolinska University Hospital, Stockholm, Stockholm, SE-141 86, Sweden; f Department of Biomedical Engineering, Groningen University, University Medical Center Groningen, Antonius Deusinglaan 1, Groningen, 9713 AW, the Netherlands

## Abstract

Reactive oxygen species (ROS) are important regulators within the ovarian follicle microenvironment and are increasingly recognized as mediators of impaired oocyte competence in assisted reproductive technologies (ART). Beyond established factors such as aging, metabolic imbalance, and environmental exposures, redox dysregulation within the follicle may contribute to variability in ART outcomes that is not explained by conventional morphological assessment. ROS are generated across multiple subcellular compartments, including mitochondria, endoplasmic reticulum, lysosomes, plasma membrane, and peroxisomes. Their compartmentalized production, together with localized antioxidant defenses, creates spatially confined redox microdomains that regulate follicular physiological activity. Disruption of this finely tuned redox balance within the follicle may result in oxidative damage affecting oocytes. Identifying oxidative stress–related biomarkers within the follicle has therefore become an attractive non-invasive approach to infer oocyte competence, as these markers may reflect integrated redox dynamics. However, accurate quantification of ROS remains challenging due to their short half-life and subcellular confinement. Most current approaches rely on measuring stable oxidative by-products or antioxidant capacity in follicular fluid or granulosa cells. However, such markers may not accurately capture follicle-specific or compartment-specific redox status, and many oxidative modifications do not directly indicate functional impairment. Although numerous biomarkers have been associated with ART outcomes, methodological heterogeneity and limited biological resolution restrict their clinical translation. Further studies using standardized methodologies and higher-resolution measurements are required to clarify their prognostic relevance.

## 1 Introduction

Infertility represents a growing global health challenge, affecting approximately 15% of couples worldwide [1,2]. Assisted reproductive technologies (ART), defined as treatments in which oocytes or embryos are manipulated outside the body [3], have transformed reproductive medicine and now account for a substantial proportion of births in many countries [3]. Despite remarkable technological progress in ART over the past four decades, live birth rates per cycle remain at approximately 30–40% [4,5]. This plateau reflects a fundamental limitation that current ART practice lacks reliable molecular indicators on oocyte competence, which is a primary determinant of embryo developmental potential [6].

Assessing oocyte quality remains one of the most critical and unresolved challenges in ART [7]. The oocyte plays a crucial role in embryo development, contributing half of the nuclear genetic material, all of the mitochondrial DNA, and essential developmental support [8]. Nevertheless, current clinical evaluation relies predominantly on morphological observation of the oocyte and surrounding cumulus cells under stereomicroscopy or inverted microscopy [9]. Such assessment is inherently subjective, poorly standardized, and lacks robust predictive value for fertilization, implantation, or live birth [7]. Moreover, oocytes and embryos that appear morphologically normal may still harbor molecular abnormalities impacting their functionality and viability [7]. Additional screening techniques, such as polarized light imaging of the meiotic spindle, have been explored in IVF laboratories, but the evidence connecting these techniques to IVF outcomes remains inconsistent and frequently lacks reproducibility [10,11].

Beyond selection limitations, current ART are largely incapable of identifying modifiable biological factors that influence oocyte competence. Among the candidate mechanisms, oxidative stress has emerged as a biologically plausible and potentially targetable contributor. Reactive oxygen species (ROS) are oxygen-derived molecules generated primarily as metabolic by-products in biological systems. Oxidative stress arises when ROS production exceeds antioxidant capacity, leading to disruption of redox homeostasis. Increasing evidence indicates that redox imbalance within the ovarian follicle may impair oocyte competence and embryo development [[12], [13], [14], [15], [16]]. Importantly, ROS are not merely cytotoxic by-products but also act as signaling mediators in folliculogenesis and meiotic progression, suggesting that dysregulated redox control may represent a modifiable contributor to ART failure. Although several studies report associations between oxidative markers and fertilization rates, embryo quality, or pregnancy outcomes, no single biomarker has demonstrated sufficient consistency or clinical validity for routine IVF practice [17,18].

In this review, we synthesize current knowledge on ROS within the ovarian follicle, critically evaluate oxidative stress–related biomarkers in relation to oocyte competence and embryo development, and discuss the methodological barriers that limit their clinical translation in ART.

## 2 Types and features of ROS

ROS can be classified into free radicals and non-radicals based on the chemical features. ROS containing unpaired reactive electrons in the outer orbit are referred to as free radicals, which mainly include nitric oxide (NO^•^), superoxide radical anion (O_2_^•-^), hydroxyl radical (OH^•^), carbonate radical anion (CO_3_^•-^), nitrogen dioxide (NO_2_^•^), and alkoxyl/alkyl peroxyl (RO^•^/ROO^•^). Hydrogen peroxide (H_2_O_2_), peroxynitrite (ONOO^−^)/peroxynitrous acid (ONOOH) and hypochlorous acid (HOCl) are considered the most important non-free radical components of ROS and do not contain an unpaired electron. Among these ROS, nitric oxide (NO^•^), superoxide radical anion (O_2_^•-^), hydroxyl radical (OH^•^) and hydrogen peroxide (H_2_O_2_) are the most common ROS in living organisms [19]. Compared to non-radicals, free radicals are generally more unstable and reactive due to the presence of an unpaired electron. Different types of ROS vary significantly in chemical features including reactivity, lifetime, ability to diffuse and preferred target. Besides, each type of ROS has its own preferred target, which can be other types of ROS, protein residues, as well as macromolecules such as DNA, RNA, protein and lipids, thereby generating downstream reactive species or leading to molecular changes [[19], [20], [21]].

## 3 Generation and elimination of subcellular ROS in ovarian follicle

**Fig. 1:**
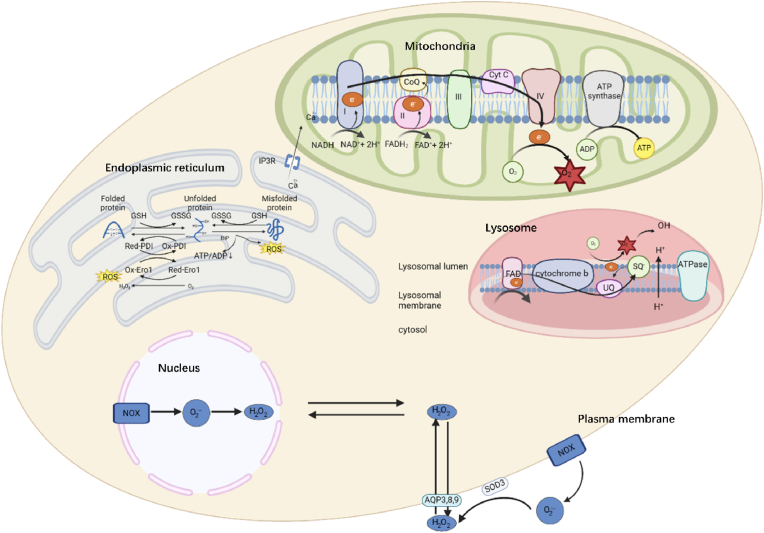
Different types of ROS is produced in cell compartments including mitochondria, lysosome, endoplasmic reticulum, nucleus and cell membrane. NOX: NADPH oxidase; GSH: glutathione; GSSG: glutathione disulfide; IP3R: inositol 1,4,5-trisphosphate receptor; Red-PDI: reduced protein disulfide isomerase; Ox-PDI: oxidized protein disulfide isomerase; FAD: flavin adenine dinucleotide; UQ: ubiquinone; SQ: semiquinone; NADH: nicotinamide adenine dinucleotide (reduced); NAD+: nicotinamide adenine dinucleotide (oxidized); FADH: flavin adenine dinucleotide (reduced); FAD+: flavin adenine dinucleotide (oxidized); CoQ: Coenzyme Q; ADP: adenosine diphosphate; ATP: adenosine triphosphate; CytC: cytochrome *c*; AQP: aquaporin; SOD3: superoxide dismutase 3.

ROS are generated through interconnected redox reactions, initiated by the reduction of molecular oxygen to superoxide (O_2_^•-^), followed by its conversion into H_2_O_2_ and other reactive species. This cascade enables rapid amplification of ROS and, if not tightly regulated, leads to oxidative stress [19,22]. To counterbalance ROS, cellular antioxidant systems, including superoxide dismutase (SOD), catalase (CAT), glutathione-dependent enzymes, and non-enzymatic antioxidants, act synergistically to maintain redox homeostasis [23,24]. Importantly, ROS generation and elimination are highly compartmentalized within cells, reflecting the distinct metabolic activities and antioxidant distributions of different organelles [25] (Fig. 1). This spatial organization is particularly relevant in the ovarian follicle, where subcellular redox balance influences oocyte competence and follicular function. Accordingly, the following sections focus on major organelle-specific sources of ROS and their regulatory mechanisms.

### 3.1 Mitochondria

Among the cellular compartments, mitochondria, the major source of ROS, have attracted the most attention. It also plays central role in ovarian follicles physiology, as its function is a determinant of oocyte quality and granulosa cell function. During oxidative phosphorylation (OXPHOS), ROS are a byproduct of ATP generation occurring at the mitochondrial electron transport chain (ETC),which consists of complex I (NADH-ubiquinone oxidoreductase), complex II (succinate-ubiquinone oxidoreductase), complex III (ubiquinol-cytochrome c oxidoreductase), and complex IV (cytochrome *c* oxidase) [26] [22]. The leakage of additional electrons from the inner mitochondrial membrane or from various oxidases then reacts with molecular oxygen to form superoxide anions (O_2_^•-^). This superoxide is the main type of ROS generated in mitochondria and considered to be a primary factor in oxidant toxicity [27]. Mitochondrial ROS concentrations are tightly regulated by multiple factors, including the redox state of the ETC and the activity of antioxidant enzymes. Further, the presence of mitochondrial DNA (mtDNA) mutations can impair ETC function and increase ROS production [28]. Additionally, mitochondrial ROS generation is influenced by cellular stressors, such as nutrient deprivation, hypoxia, and exposure to environmental toxins [29]. Notably, a recent milestone study demonstrated that human and Xenopus oocytes maintain ROS-free mitochondrial metabolism by suppressing complex I activity, providing an explanation for why patients with complex I–related mitochondrial defects do not exhibit subfertility [30]. Furthermore, follicle-stimulating hormone (FSH), a key regulator of granulosa cell proliferation, has been shown to preferentially enhance mitochondrial ROS production rather than cytoplasmic ROS generation, highlighting the compartment-specific regulation of ROS in granulosa cells [31].

Although mitochondrial ROS have been extensively studied in the context of ovarian follicle physiology, investigations linking mitochondrial ROS–related biomarkers to ART outcomes remain limited. This limitation is partly attributable to the intrinsic properties of ROS species, particularly superoxide, which is highly unstable and has a very short half-life, making it difficult to detect reliably [[19], [20], [21]]. Current detection approaches for mitochondrial ROS, such as Mitotracker, are predominantly dye-based and are subject to technical constraints such as photobleaching and limited specificity [32]. In addition, these methods typically provide bulk measurements, lacking the resolution to capture heterogeneity at the level of individual oocytes, which may differ substantially in their redox status.

### 3.2 Endoplasmic reticulum (ER)

The endoplasmic reticulum (ER) has been extensively studied in both granulosa cells and oocytes [33,34], primarily in the context of ER stress. ER stress, characterized by the accumulation of unfolded or misfolded proteins, arises under various physiological and pathological conditions, including oxidative stress, which either increases the demand for protein folding or impairs the folding capacity of the ER [35]. In addition to being a target of oxidative stress, the ER also functions as a source of ROS [36]. During oxidative protein folding, the formation of disulfide bonds in newly synthesized proteins produces ROS as a byproduct, and the accumulation of misfolded proteins can further enhance ROS generation via ER-associated degradation (ERAD) [14]. In addition, calcium release from the ER can activate NADPH oxidases (NOX), thereby promoting ROS production [37]. Notably, oxidative stress and ER stress are closely interconnected, forming a feedback loop in which ROS generation induces ER stress, which in turn further increases ROS production [38]. Current research on ER-related ROS in ovarian follicles is largely derived from animal studies. Increased intracellular ROS associated with aging can damage the ER and disrupt Ca^2+^ homeostasis, leading to enhanced fragmentation in porcine MII oocytes [39]. Conversely, antioxidants such as melatonin have been shown to improve meiotic maturation of porcine oocytes by alleviating ER stress during in vitro maturation [40].

### 3.3 Lysosomes

The role of lysosomes in ovarian follicles has been increasingly recognized. Although lysosomes are not prominent within the oocyte itself, they play essential roles in follicular atresia, ovulation, luteal regression, luteal cell survival, and ovarian steroidogenesis [41], processes that are closely associated with the regulation and balance of ROS.

Lysosomes are also capable of generating ROS. Experimental evidence from mouse hippocampal cell lines indicates that lysosomes, in addition to mitochondria, serve as sites of basal ROS generation [42]. An electron transport chain has also been identified within lysosomes, with hydroxyl radicals (OH•) representing a major ROS species produced in this compartment [43]. However, despite these observations, direct evidence linking lysosomal ROS to ovarian follicle physiology remains lacking.

### 3.4 Plasma membrane

The plasma membrane represents an important site of ROS generation involved in cell signaling [44]. This process is primarily mediated by NADPH oxidases (NOX), a family of membrane-localized enzymes that transfer electrons from intracellular NADPH across the membrane to molecular oxygen, generating superoxide (O2•^-^) and subsequently hydrogen peroxide (H_2_O_2_). These ROS can be produced extracellularly and re-enter the cell through channels such as aquaporins, thereby participating in intracellular signaling pathways. Emerging evidence suggests that NOX-derived ROS in mature ovarian follicles may have a conserved physiological role in regulating follicle rupture and ovulation [45]. In patients with polycystic ovary syndrome (PCOS), enhanced NADPH oxidase activity at the plasma membrane contributes to increased ROS production and the maintenance of a chronic inflammatory state [46].

However, current evidence linking NOX-derived ROS to ovarian function is largely derived from experimental or disease-associated studies, and direct causal relationships in human follicular physiology remain insufficiently established. Furthermore, the extracellular generation of ROS by NOX poses additional challenges for accurate measurement, as current approaches often lack the spatial resolution required to differentiate between extracellular and intracellular ROS signaling. These limitations hinder a precise understanding of the role of NOX-derived ROS in follicular development and ART outcomes.

### 3.5 Peroxisomes

Peroxisomes are multifunctional organelles that both generate and detoxify reactive ROS, thereby playing a dual role in cellular redox homeostasis. They represent a major site for both ROS production and clearance, largely due to the presence of ROS-generating oxidases and antioxidant enzymes. Among these, catalase which localized in the peroxisomal matrix, is a key detoxifying enzyme that prevents the accumulation of H_2_O_2_ within cells [47]. Peroxisomes also contain enzymes capable of generating H_2_O_2_, O2•-, and •OH. The presence and activity of peroxisomes in ovarian cells have been well documented, with suggested roles in follicular growth, oocyte maturation, ovulation, and steroidogenesis [48]. More recently, peroxisomes have been identified in all follicular cell types, including the oocyte, with their abundance and protein composition shown to vary according to follicular stage, estrous cycle, and cell type [49]. Given that peroxisomal ROS metabolism is largely mediated by enzymatic reactions, current approaches to assess peroxisome-related ROS are primarily based on measuring the levels or activity of these enzymes. However, such indirect measurements may not accurately reflect real-time ROS dynamics, as enzyme abundance does not necessarily correlate with ROS production or scavenging capacity under physiological conditions.

## 4 Dual role of ROS in ovarian follicles: physiological and pathological

**Fig. 2:**
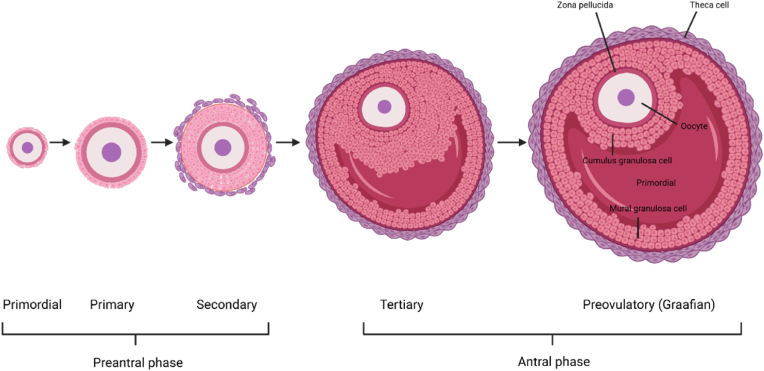
Ovarian follicles in the human ovary are characterized by a long period of development, with follicles at various stages, from primordial to preovulatory size.

Ovarian follicle is the functional units governing female reproductive lifespan [50]. Each follicle contains an oocyte surrounded by granulosa and theca cells and undergoes tightly regulated developmental progression from primordial to preovulatory stages [51,52](Fig. 2). Throughout this process, coordinated cellular interactions and endocrine signaling within the follicular microenvironment support oocyte growth and maturation [[53], [54], [55]]. As the immediate microenvironment of the oocyte, the ovarian follicle provides the structural and metabolic context in which ROS are generated and regulated.

Physiologically, the antral follicle is exposed to low O_2_ concentrations varying from 1% to 4% [56]. This hypoxia condition induces ROS generation. A physiological level of ROS is essential in regulating folliculogenesis, meiosis and ovulation by acting as secondary messengers for cellular signaling [57]. For instance, GC ROS generation, serving as an intracellular messenger or signal transducer for follicular angiogenesis—a critical process for follicular growth, with its disruption potentially leading to follicular atresia [58,59]. Spontaneous resumption of meiosis in rat oocyte-cumulus complexes was found to be inhibited by addition of antioxidants in vitro [60]. In addition, ROS is considered as a critical inducer of ovulation, as increased ROS concentrations in the preovulatory follicle are observed before ovulation which induces oocyte meiotic maturation [61]. During the luteal phase, a rapid increase of ROS due to the increase in serum LH concentrations or PGF2α were present in the rat corpus luteum [62]. Superoxide radicals, in turn, regulate intracellular progesterone secretion in the rat corpus luteum [63]. An involvement of ROS in corpus luteum regression has also been reported, as ischemia-reperfusion-induced ROS production, rather than prostaglandins, inhibited rat corpus luteum function [64]. A physiological level of ROS can be also positively associated with favorable ART outcome. Conception cycles were linked to serum with a higher tendency for oxidation compared to non-conception cycles [65].

However, ROS is a double-edged sword and overabundant ROS are presumably pathological to ovarian follicles. Excessive ROS production is associated with a wide range of conditions such as prolonged hypoxia or hyperoxia, toxins and some lifestyle factors such as smoking [66]. Prolonged exposure to hypoxia can cause oxidative stress [67]. A significantly low oxygen content in human follicular fluid is linked to reduced oocyte developmental potential such as a higher occurrence of cytoplasmic defects, impaired cleavage, and abnormal chromosomal segregation in human oocytes [68]. On the other hand, sustained hyperoxia conditions can also induce oxidative stress. A recent study found that follicle viability and quality in the human ovarian cortex were lower following in vitro culture with high O_2_ tension (20%) compared to low O_2_ tension (5%). Follicle apoptosis and senescence rates, as well as GC oxidative stress and DNA double strand break rates were also reduced in the low O_2_ tension group than in the high one [69]. The effects of excessive ROS on ovarian follicles vary among different follicular developmental stages. Ovarian follicles exposure to di (2-ethylhexyl)phthalate (DEHP) exposure can induce oxidative stress, leading to oocyte cyst breakdown and inhibition of primordial follicle formation in newborn mice [70]. The number of primordial follicles and antral follicles was significantly decreased in pup mice that were exposed to DEHP during the lactation period [71]. Inhibited antral follicle growth and a reduced number of large follicles were also observed after 20-day exposure to DEHP during the pre-pubertal stage in mice [72]. Lifestyle factors such as smoking primarily exert adverse effects on pre-ovulatory ovarian follicles, an impact that is partly mediated by excessive ROS exposure [73]. More examples on the association between oxidative stress and ovarian follicle component, including oocytes, GCs, theca cell layers and FF are discussed in detail below.

### 4.1 Oxidative stress and the oocyte

Located within the ovarian follicle, the oocyte relies on the surrounding follicular fluid and cumulus cells for oxygen supply [56]. A decreased antioxidant status, leading to an increase in oxidative stress, such as observed e.g. in obesity, has been suggested to accompany a decline in oocyte quality [74,75]. To date, oxidative stress has been found to adversely affect the oocyte in terms of meiotic progression, spindle integrity, chromosomal aneuploidy, DNA damage, apoptosis and cytoskeleton structure [76]. Tamura et al. found that oxidative stress can inhibit mouse oocyte meiotic maturation, which can be prevented by melatonin, a free radical scavenger [77]. Tarin et al. revealed that oxidative stress leads to aneuploidy and changes in spindle morphology in mouse oocytes when exposed to an oxidizing agent. A decreased incidence of oocyte aneuploidy was observed in response to antioxidant supplements in mice [78]. Repeated ovarian stimulation with gonadotropins induced mitochondrial DNA mutations and oxidative DNA damage in mouse oocytes [79], probably due to the significantly increased occurrence of spindle defects and resulting in chromosomal errors [80]. A previous study using follicles from primary to pre-antral stages isolated from adult human ovaries demonstrated that H_2_O_2_- induced oxidative stress can promote oocyte apoptosis and necrosis in resting follicles in a dose- and time-dependent manner [81]. Furthermore, advanced glycation end products (AGEs) accumulation upon oxidative stress can induce protein damage and inflammation, thereby inhibiting oocyte developmental competence [82,83]. Although oxidative stress is closely related to oocyte quality, biomarkers that measure ROS levels directly at human oocytes are currently unavailable in clinical practice due to ethical considerations and potential associated harm.

### 4.2 Oxidative stress and GCs

During follicle growth, rapid GC proliferation results in increased energy and nutrient demand due to metabolic rate acceleration, leading to increased ROS production and oxidative stress [84]. Obesity or lifestyle factors such as alcohol, also induce oxidative stress in GCs [85]. Oxidative stress can negatively affect GCs in a variety of different ways. It induces programmed GC death, such as apoptosis, which leads to follicular atresia [86]. FSH remarkably attenuates the oxidative stress induced decline in viability of GCs by restraining autophagy both in vitro and in vivo, thereby maintaining cellular integrity and function of GCs [84]. In mice, enhanced ROS levels induced GC senescence, which can be delayed by antioxidants [87]. However, it is worth noting that there are two major subtypes of GCs (cGC and mGC) with significant spatial and temporal heterogeneity. One of the key functions of cGCs is to shield the oocyte against oxidative stress [88]. cGCs and mGCs exhibit different functions and gene expressions [89,90] A recent study applying diamond relaxometry, a novel technique that is able to probe free radical generations on subcellular levels, shows that cGC and mGC exhibit different responses to oxidant [91]. Therefore, separating cGCs and mGCs could conceivably reveal added information when investigating human GC samples.

### 4.3 Oxidative stress and theca cell layer

The theca cell layer, consisting of the theca interna and theca externa, plays a critical role in ovarian folliculogenesis and steroidogenesis [92]. The steroidogenic cells of the theca interna express luteinizing hormone receptor (LHR) and possess the enzymes required for production of androgens, which are subsequently converted into estrogens by GCs [93]. ROS are generated as a byproduct and act as signaling molecules at physiological levels in this process, as well as in theca cell proliferation and differentiation [92]. On the other hand, theca cells possess endogenous antioxidant mechanisms, such as catalase. Its activity is increased during ovarian development and luteinization in rats [94]. However, under conditions such as ageing, environmental toxins, and metabolic disorders, excessive ROS can be produced or antioxidant defenses can be overwhelmed, leading to oxidative stress in theca cells. Oxidative stress, in turn, significantly impacts the function of theca cells. Studies have shown that oxidative stress can disrupt steroidogenesis in theca cells, impairing androgen production by altering the expression and activity of key steroidogenic enzymes such as cytochrome P450 enzymes, thereby reducing androgen synthesis [95,96]. Moreover, oxidative stress can induce apoptosis in theca cells, further compromising follicular integrity and function [97]. Future research to understand the intricate balance between ROS and antioxidant defenses in theca cells is crucial for developing therapeutic interventions aimed at mitigating oxidative stress-induced ovarian dysfunction [66].

### 4.4 Oxidative stress and follicular fluid

Follicular fluid originates from multiple sources within the ovarian follicle and subsequently displays a dynamic composition. It primarily comes from plasma filtered through thecal capillaries within the theca interna, the outer layer of cells surrounding the follicle. The capillaries in the theca interna allow plasma to pass through, contributing to the formation of follicular fluid [98]​. GCs also actively secrete various substances, including steroid hormones, proteins, enzymes, growth factors, and other signaling molecules that are crucial for oocyte maturation and follicular development into the follicular fluid [98]. During follicle growth, follicular fluid provides nutrition for oocyte development, thereby affecting oocyte quality. In addition, follicular fluid contains a variety of antioxidants, such as vitamin C that may potentially provide buffer capacity to allow the oocyte to only be exposed to optimal levels of ROS. A study investigating 145 women who attended an in-vitro fertilization program revealed a lower rate of lipid peroxidation (<1.5%) in the preovulatory follicular fluid compared to that in serum, indicative of the presence of an efficient antioxidant defense system in follicular fluid surrounds the oocyte [99]. In addition, hormones such as FSH and estrogen [100], metabolites such as bile acids and trimethylamine-N-oxide [101], either from the systemic circulation or locally from GCs/oocytes, are present in the follicular fluid and were reported to regulate redox status. It is thus conceivable that follicular fluid not only contributes to the redox balance in ovarian follicles but may also reflects an overall redox status of the follicles. In the context of research, considering the easy access and “do no more harm” during IVF procedure, follicular fluid can potentially serve as an important biological sample indicative of oocyte health.

## 5 Current knowledge of oxidative stress-related biomarkers in IVF practice

A biomarker, short for “biological marker,” is an objectively measured characteristic that indicates normal biological processes, pathogenic processes, or responses to therapeutic interventions. It includes everything ranging from measurable medical signs, which is distinct from symptoms perceived by patients to complex molecular interactions [102]. Biomarkers are integral to modern clinical and research practices, where their relationship to relevant clinical endpoints is a focus of ongoing refinement [102]. The most promising biomarkers are those that exhibit a strong correlation with the disease's pathophysiological processes [103]. The significant role of ROS in follicle pathophysiology, as reviewed above, has led to ROS quantification as a promising biomarker. The accessibility of granulosa cells (GC) and follicular fluid, which are typically discarded after oocyte retrieval, makes these samples strong candidates for biomarker analysis. Since only approximately 16 % of all oocytes aspirated for in vitro fertilization (IVF) develop into a normal embryo that leads to a birth [104], a search for improved biomarkers in the follicle micro-environment for oocyte quality based on ovarian cellular signaling and metabolism is an emerging topic of high clinical relevance.

**Fig. 3:**
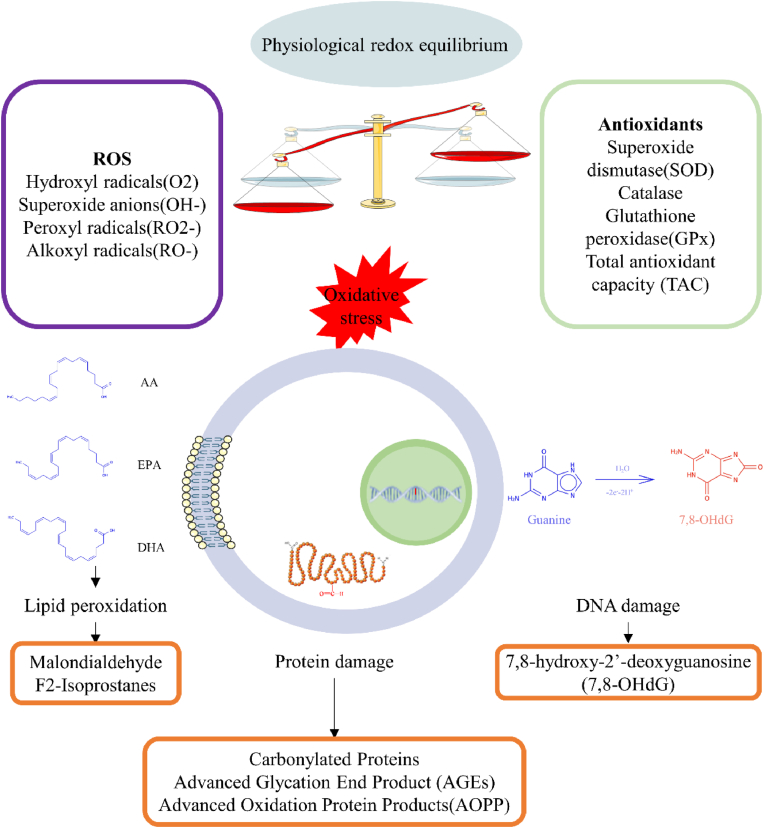
Classical oxidative stress biomarkers include direct ROS measurement, ROS-induced lipid/DNA/protein modifications and ROS generating enzyme, as well as antioxidant.

Here, we summarize current knowledge on the most relevant oxidative stress biomarkers in IVF clinical practice, as well as their detection methods with advantages and disadvantages. Oxidative stress biomarkers for clinical biological samples are categorized as direct ROS measurement, oxidative damage assessments, which include ROS-induced lipid/DNA/protein modifications, as well as measurement of antioxidants, which can be enzymatic or non-enzymatic (Fig. 3)

### 5.1 Direct ROS measurement

Direct ROS detection methods measure the signal coming directly from the ROS. Although electron spin resonance (ESR) has been considered as the golden standard for direct measurement of free radical species, this technique has never been applied in ART practice. A typical example of the direct ROS measurement in follicular fluid and GCs is the use of the DCFH-DA (dichlorodihydrofluorescein diacetate) for intracellular ROS measurement. DCFH-DA enters the cells readily and is oxidized to the highly fluorescent product dichlorofluorescein (DCF) nonspecifically by several ROS. Thus, DCFH-DA (similarly to most ROS dyes) is not specific for any particular ROS. ROS levels measured with DCFH-DA in GCs was negatively linked with IVF parameters including metaphase II oocyte rate and ongoing pregnancy rate [105]. The chemiluminescence assay provides another direct method for measurement of both extracellular and intracellular ROS, utilizing luminol as the probe [106].

It detects oxidative end products generated by an in-vitro reaction between ROS and specific reagents, producing light that is measured with a luminometer. This assay has demonstrated high reliability and accuracy as diagnostic tools for detecting ROS in human semen [[106], [107], [108]]. Follicular fluid ROS measurement by chemiluminescence among women undergoing IVF/ICSI showed in a majority of studies significant correlations between follicular fluid ROS levels and IVF parameters including number of oocytes retrieved, oocyte quality, fertilization rate, embryo quality and pregnancy [[109], [110], [111], [112], [113], [114]] (Table 1). Inconsistent results were also reported by a cross-sectional study among PCOS patients: no significant association between follicular fluid ROS and any of the clinical parameters [115]. The variability in results across different studies can be attributed to several factors, including differences in the concentration of reactants, sample volume, reagent injection methods, temperature control, and background luminescence [108]. These variables can significantly influence the reaction dynamics and, consequently, the measurement outcomes.

**Table 1:** Studies on the association between ART outcomes and oxidative stress-related biomarker through direct ROS measurement.

Biomarker	Sample	N	Population (Inclusion criteria)	Exclusion criteria	Ovarian stimulation	Method	Positive findings	Negative findings	Reference
ROS	FF	100	PCOS (n = 43), tubal (n = 57)	endometriosis, male factor, unexplained infertility, diminished ovarian reserve	GnRH antagonist (PCOS); varying (tubal)	CL	N.A.	oocytes retrieved, fertilization, cleavage, embryo quality, pregnancy, miscarriage	[115]
ROS	FF	48	Tubal, 26-40 y/o partners/donors with normozoospermia and optimal ROS levels	contaminated with blood/media; more than one oocyte/no oocytes	long protocol	CL	oocyte quality, fertilization, embryo quality	N.A.	[109]
ROS	FF	189	IVF	N.A.	long protocol	CL	mature oocyte, cleaved embryos	N.A.	[110]
ROS	FF	138	ICSI	N.A.	long protocol	CL	oocytes retrieved, pregnancy	N.A.	[111]
ROS	FF	340	Endometriosis (n = 200), tubal (n = 140) 26-40 y/o BMI<25 kg/m^2^; infertility duration >2 years	N.A.	long protocol	CL	Mature oocyte, embryo quality, pregnancy	N.A.	[112]
ROS	FF	78	tubal	gross hydrosalpingeal changes, dense pelvic adhesion because of endometriosis or PID	long protocol	CL	embryo quality	oocyte maturity	[113]

ROS: reactive oxygen species; PCOS: polycystic ovarian syndrome; FF: follicular fluid; CL: chemiluminescence; PID: pelvic inflammatory disease; cGC: cumulus granulosa cell; N.A.: not applicable.

### 5.2 Assessment of oxidative damage

When ROS formation exceeds elimination, oxidative stress arises, leading to damage to cellular components, including DNA/RNA, proteins, and lipids [116]. Specifically, excessive hydroxyl radicals and peroxynitrite can damage cell membranes and lipoproteins through lipid peroxidation, producing cytotoxic and mutagenic compounds such as conjugated dienes and malondialdehyde (MDA) [117]. Oxidative stress can also induce conformational modifications of proteins, impairing their enzymatic activity (e.g., advanced oxidation protein products, AOPP), and cause oxidative DNA lesions, such as 8-hydroxy-2′-deoxyguanosine (8OHdG), which may result in mutations [117]. Assessment of oxidative damage, therefore, focuses on detecting these degradation products, such as lipid, protein, and DNA modifications caused by ROS [118]. These assessments are stable and less technically challenging compared to direct ROS measurement. However, they provide qualitative rather than quantitative information about ROS generation levels, as they do not account for increased radical production that has not yet caused detectable cellular damage [118]. Studies on the association between ART outcomes and oxidative stress-related biomarker through oxidative damage assessment are summarized in Table 2.

**Table 2:** Studies on the association between ART outcomes and oxidative stress-related biomarker through oxidative damage assessment.

Biomarker	Samples	N	Population (Inclusion criteria)	Exclusion criteria	Ovarian stimulation	Method	Positive findings	Negative findings	Reference
8-OHdG	FF	54	Nonsmokers; free from major medical illness	myoma, adenomyosis, congenital uterine anomaly, ovarian tumors estrogen/progesterone/androgen use chronic use of any medication	long protocol	ELISA	degenerate oocytes	N.A.	[77]
8-OHdG	FF	117	IVF	N.A.	long agonist (n = 38); short agonist (n = 4) antagonist (n = 36)	ELISA	fertilization (ICSI), good quality blastocysts	Fertilization (cIVF), goodquality embryo, blastocysts	[17]
8-OHdG	mGC/cGC	96	Endometriosis (n = 17), male factor (n = 17), tubal factor (n = 29), unknown (n = 33)	N.A.	long protocol	immunocytochemical staining	FR (cGC/mGC), HQR (cGC/mGC)	N.A.	[119]
Protein carbonyl	FF	82	aged 24–39 for IVF	history of thrombosis, hypertension, diabetes, or any metabolic disorder. medication (eg: cholesterol lowering vitamin supplements	short protocol	ELISA	pregnancy	N.A.	[120]
AGE	FF	127	ovarian dysfunction, tubal, idiopathic, male, both male and female	PCOS, liver, kidney, heart or blood vessel diseases, untreated or insufficiently corrected endocrinopathies	long protocol	ELISA	NOR, NFO, NHE, FR, HQR	N.A.	[121]
AGE (pentosidine)	FF	157	ovarian dysfunction (n = 52), tubal factors (n = 19), endometriosis (n = 7), male factors (n = 3), ovarian dysfunction plus male factors (n = 49), tubal plus male factors (n = 18), unexplained (n = 9)	uterine factors	long protocol	ELISA	FG, NOR, NFO, NHE, OPR	N.A.	[122]
AGE (glycer-AGE)	FF	157	indicated as above∗	uterine factors	long protocol	ELISA	FG, NOR, NFO	NHE, OPR	[122]
AGE (carboxymethyl lysine)	FF	157	indicated as above∗	uterine factors	long protocol	ELISA	FG, NOR, NFO, NHE	OPR	[122]
8-IP	FF	100	PCOS (n = 43), tubal (n = 57)	endometriosis, male factor, unexplained infertility, and diminished ovarian reserve	GnRH antagonist (PCOS); varying (tubal)	ELISA	Miscarriage (PCOS)	Miscarriage (tubal) NOR, fertilization, CR, grading of embryos	[115]
MDA	FF	41	male factor (n = 16), tubal factor (n = 17), idiopathic (n = 3), ovulatory factor (n = 5)	N.A.	long protocol	TBA	pregnancy rate	oocyte maturity, FR, CR, embryo quality	[123]
MDA	FF	48	tubal factors, 26-40 y/o partners/donors with normozoospermia and optimal ROS levels	contaminated with blood/media; more than one oocyte/no oocytes	long protocol	TBA	oocyte quality, FR, embryo quality	N.A.	[109]
MDA	FF	253	1 ^st^ IVF cycle	structural abnormality, spontaneous conception , sperm abnormality, fallopian tube pathology, endometriosis undergoing.	N.A.	N.A.	Viable pregnancy	N.A.	[124]
MDA	FF	203	IVF	systemic disease, uterine anomalies, endometrial path-ology	long agonist, short agonist, antagonist	N.A.	pregnancy	NOR	[125]
MDA	FF	80	norm-responder for IVF	endocrinopathy, hyperandrogenism, PCOS, systemic disease	long agonist (n = 58), short agonist (n = 8), antagonist (n = 14)	TBA	N.A.	NOR, FR	[126]
MDA	FF	340	Endometriosis (n = 200), tubal (n = 140) 26-40 y/o BMI<25 kg/m^2^; infertility duration >2 years	N.A.	long protocol	TBA	pregnancy	N.A.	[112]
MDA	FF	78	tubal factors	gross hydrosalpingeal changes, dense pelvic adhesion because of endometriosis or PID	long protocol	TBA	embryo quality	oocyte maturity	[113]
MDA	cGC	102	IVF	<23 or > 40 y/o, diabetes	antagonist	TBA	NOR	N.A.	[127]

8-OHdG: 8-hydroxy-2¢-deoxyguanosine; 8-IP: 8-Isoprostane; MDA: malondialdehyde; AGE: Advanced glycation end product; FG: follicle growth; NOR: number of oocytes retrieved; NFO: number of fertilized oocytes; NHE: number of high-quality embryos; FR: fertilization rate; CR: cleavage rate; HQR: high quality embryo rate; OPR: ongoing pregnancy rate; CL: chemiluminescence; FF: follicular fluid; cGC: cumulus granulosa cell; PCOS: polycystic ovarian syndrome; TBA: thiobarbituric acid; N.A.: not applicable.

#### 5.2.1 Markers of lipid peroxidation

Lipid peroxidation is generally recognized as a process in which high levels of oxidants attack lipids that contain carbon-carbon double bonds. Isoprostanes and MDA are two of the most widely investigated lipid peroxidation biomarkers.

##### 5.2.1.1 F2-isoprostanes

The isoprostanes are a unique family of prostaglandin-like compounds formed in vivo involving the ROS-initiated peroxidation of arachidonic acid, which is particularly enriched in phosphoilipids of biological membranes [128]. Among this family, F2-Isoprostanes are a specific class that contains F-type prostane rings and are widely regarded as the gold standard biomarker for assessing endogenous oxidative stress due to their stable and robust characteristics [128]. After being released from the cell membrane into circulation, isoprostanes can be detected and quantified in blood, tissues and urine. Their concentration is mainly determined by the production rather than metabolism or excretion, thus allowing them to be more reflective of oxidant stress in vivo [129].

The application of isoprostanes as a biomarker for IVF outcomes has been explored in serum [130], urine [131] and follicular fluids [115,132]. Younis et al. found no correlation between serum isoprostane and pregnancy in 15 women undergoing intrauterine insemination (IUI) or IVF [130]. Interestingly, in a prospective cohort study which included 481 women following IVF/IUI treatment, a moderate degree of oxidative stress (middle tertile), measured as isoprostane metabolite 2,3-dinor-5,6-dihydro-15-F2t-isoprostane (F2-Isoprostane-M) in urine, was recently found to have a higher fertilization rate compared to the women with urinary F2-Isoprostane-M level in the upper (0.66 [95% CI: 0.61, 0.71]) and lower (0.69 [95% CI: 0.64, 0.73]) tertiles [131]. Although the study did not adjust for number of follicles, it lends evidence to the idea that a certain threshold of oxidative stress may be necessary for physiological reproductive processes and antioxidant approaches to attenuate cellular oxidative damage should be carefully balanced. Moreover, the F2-IsoP-M measured in this study, is a major metabolite of free 8-iso-prostaglandin F2α (8-iso-PGF2α) and minimally produced from the kidney, thus making it a more sensitive and reflective biomarker of systemic oxidative stress, compared to the traditionally used 8-iso-PGF2α, which is renal-generated and excreted [133].

A previous study demonstrated similar 8,12-iso-iPF2α levels between follicular fluid and plasma [132]. The association between F2-isoprostane level and IVF parameters has also been explored in the follicular fluid of women undergoing IVF [115,132]. In women with PCOS, higher median 8-isoprostane was observed in those who had a miscarriage, while no correlation was found in the tubal factor infertility group [115]. This suggests that the predictive value of follicular fluid isoprostane for pregnancy should be further explored in the context of different infertility groups based on etiology.

Although cheap and user-friendly commercial immunoassay kits for isoprostanes have been developed, problems such as variable performance and poorly correlated results with mass spectrometric techniques hinder their applications; mass spectrometric techniques represent the gold standard for isoprostane quantification [134].

##### 5.2.1.2 Malondialdehyde (MDA)

MDA is an end-product following decomposition of arachidonic acid and larger polyunsaturated fatty acids and is widely used to evaluate the degree of peroxidative cell damage [135]. The thiobarbituric acid (TBA) assay is the most frequently used colorimetric method for quantifying the extent of membrane lipid peroxidation in vitro [136]. The strength of the method is that it is suitable for high throughput analysis. However, there is a lack of specificity for MDA, since compounds that absorb in the same range are produced by reactions between TBA and aldehydes instead of MDA [137]. Some commercially available ELISA kits for MDA detection have already shown favorable performances with improved specificity [138]. With these methods, the levels of MDA in systemic and local samples, including GCs and follicular fluid, have been explored in the context of a variety of reproductive parameters in a number of clinical studies. The application of MDA as a biomarker for IVF outcomes has been explored in serum [139,140], follicular fluid [109,112,113,124,125] and cumulus GCs [127]. Moderate systemic oxidative stress, measured as serum MDA had no detrimental effects on live birth rate in PCOS patients undergoing IVF/ICSI treatment [139]. In fact, the MDA levels were even positively linked to the number of good-quality embryo [139]. These studies further highlight the key role of physiological ROS levels in embryo development and occurrence of conception in IVF. In contrast, when compared to patients with normal oxidative status, patients with increased serum MDA levels (higher than 75 percentile) had a lower pregnancy rate and a higher miscarriage rate [140]. However, this study did not correct for ovarian response, so there exists a potential bias that the estradiol levels are also high due to high oocyte yield, thus affecting the embryo-endometrial asynchrony, an issue that should be cautiously taken into account in future studies.

With a lipid-rich membrane, oocytes are vulnerable to the adverse effects of ROS and lipid peroxidation [141]. Due to the high lipid concentration in oocytes, MDA levels are suitable biomarkers. These can be tested either in GCs or in follicular fluid, to be associated with oocyte oxidative stress status. Several studies have demonstrated significant negative correlations between MDA levels in follicular fluid and IVF outcome parameters including oocyte retrieval, oocyte maturation rate, fertilization rate, embryo quality and pregnancy rate [109,112,113,124,125]. Consistently, research on cumulus cells also indicated significantly decreased MDA levels in high responders to hyperstimulation IVF [127]. However, it is still rather difficult to draw a clear conclusion on the association between MDA and IVF parameters, since contrasting evidence showing no effect of FF MDA levels on ovarian response, fertilization rate and embryo quality was also reported [123,126]. This can be the consequence of differences in the characteristics of patients included (high responder vs normal responder) and variations in the samples (cGC vs FF) tested [142].

#### 5.2.2 Markers for DNA oxidation

##### 5.2.2.1 8-Oxo-7,8-dihydro-2′-deoxyguanosine (8OHdG)

DNA oxidative modification serves as a common biomarker of oxidative damage by measuring the oxidation of guanine to 8-oxo-7,8-dihydro-2′-deoxyguanosine (8OHdG, or 8-oxodG). The application of 8OHdG as a biomarker has been studied for cumulus/mural GCs and follicular fluid in IVF practice. Seino et al. found a negative association between the fertilization rate as well as embryo quality (determined by fragmentation of the embryo mass) and 8-OHdG indices both in cumulus and mural GCs, which were measured by immunocytochemical staining [119]. Consistently, investigating the follicular fluid 8OHdG using ELISA kits, studies from Tamura et al. (N = 54) and Nishihara et al. (N = 81) observed that 8OHdG levels were negatively associated with oocyte quality, fertilization rate and rate of good quality blastocysts [17,77]. However, according to a recent published guideline, ELISA kits should be cautiously used due to their low sensitivity and specificity and the batch effects. Besides, cross-reaction between 8-hydroxyguanosine (8OHG) and 8OHdG may also exist [21]. The presently best methodologies to measure 8OHdG and 8OHG, according to this guideline, remains the Comet assay which uses DNA repair enzymes, for cell samples and the ultra-performance LC–MS/MS for body fluids [21]. A study applying Comet assay showed that there was no correlation between cGC DNA damage and ICSI outcome (fertilization and embryo quality) [143]. Therefore, the link between DNA damage and IVF outcomes remains inconclusive.

#### 5.2.3 Markers for protein oxidation

##### 5.2.3.1 Protein carbonyl

A variety of proteins and amino acid residues can serve as targets for ROS, which modifies amino acid residues resulting in cross-linking, conformational changes and protein function loss of proteins. There is a large body of literature implicating the accumulation of oxidatively damaged proteins in a variety of pathologies such as diabetes and neurodegenerative diseases [144,145]. Based on the rationale that carbonyl groups are introduced into the side chains of proteins during protein oxidation, the measurement of protein carbonyl is reflective of oxidative protein damage. In fact, protein carbonyl has been the most widely used biomarker since no special equipment is needed. Protein carbonyl can thus be measured in most biochemistry laboratories [146]. Meanwhile, it offers the advantage of relatively early formation and stability, as well as many highly sensitive methods available, including ELISA, or spectrophotometric assays to detect its derivative after modification [147]. Nonetheless, none of these methods provide information on the extent of oxidative damage to a specific protein in a sample. This seems to be a common drawback of oxidative damage measurement [146].

A study recruiting 82 patients undergoing IVF-ET has analyzed this biomarker in follicular fluid using ELISA and found that there was a 2-fold increase of protein carbonyl groups in non-pregnant females compared to those who get pregnant [120]. However, it should be noted that protein oxidation products do not necessarily contain carbonyls. Thus, additional approaches using fundamentally different principles to quantify individual oxidation products, such as using specific and validated antibodies, are more recommended according to the newly published guideline [21].

##### 5.2.3.2 Advanced oxidation protein products (AOPP)

Proteins can also react with chlorinated oxidants by chlorination of amino acid residues, leading to generation of 3,5- dichloro-tyrosine and 3-chloro-tyrosine (3-Cl-Tyr) as main oxidation products, which are generally termed as AOPP. By using spectrophotometry, the level of AOPP can be measured to reflect the degree of protein damage in oxidative stress [148]. In a study including 64 women with tubal infertility, the follicular fluid AOPP level was inversely correlated with the proportion of mature oocytes, fertilization rate, cleavage rate and good embryo rate [149].

##### 5.2.3.3 Advanced glycation end products (AGEs)

AGEs are non-enzymatic glycation reaction product of reducing sugars with amino groups of proteins or lipids. The generation and accumulation of AGEs, which may lead to carbonyl stress, is believed to contribute to ovarian aging by inducing DNA damage, spindle aberrations and affecting mitochondrial integrity in oocytes [150]. By binding with its specific receptors AGE RAGE that are expressed in granulosa-lutein cells, increased levels of AGE in response to oxidative stress activate NADPH oxidase, which in turn exacerbates oxidative stress [151]. This vicious cycle then serves as one of the critical molecular mechanisms underlying GC senescence and ovarian aging [151]. Based on their fluorescent properties, AGEs can be measured either by applying spectrofluorometric methods or using specific antibodies [152]. Researchers have found that steroidogenic gene expression and estradiol (E2) release in human cumulus GCs were significantly increased when treated with AGE precursors, and the effects can be partially reversed by vitamin D3 [153]. Follicular fluid AGEs concentration has also been measured among 127 women undergoing IVF/ICSI with a GnRH agonist protocol. The researchers found that follicular fluid AGE concentration, independent of age, was inversely correlated with number of retrieved and fertilized oocytes, fertilization rate and high-quality embryo rate [121]. In this study, women with PCOS were excluded since increased serum levels and stronger GC localizations of both AGEs and their receptors were observed in women with PCOS [154,155]. Interestingly, another study that recruited 157 patients with PCOS while excluding uterine factors has revealed consistent findings [122]. Three kinds of AGEs including glyceraldehyde-derived AGE, pentosidine and carboxymethyl lysine in follicular fluid were all negatively correlated with follicle growth (numbers of follicles larger than 12 mm in diameter), numbers of retrieved and fertilized oocytes and embryos. Specifically, lower levels of follicular fluid pentosidine is predictive of ongoing pregnancy, in addition to the two conventional determinants (age and Day-3 FSH) [122]. Thus, in addition to the widely accepted consensus that AGEs are critically relevant in the aspects of GC dysfunction, abnormal ovarian histology and systemic metabolic dysregulation in PCOS, the co-occurrence of ovarian dysfunction and high AGEs levels in women without PCOS suggests that AGEs may in fact, be contributors in folliculogenesis instead of only PCOS pathology [156].

### 5.3 Anti-oxidant measurement

Human oocytes have natural antioxidant defenses, either enzymatic or non-enzymatic, provided by its surrounding follicular environment. A study investigated metaphase II mouse oocytes in vitro and found that the defense against oxidative stress of oocytes mainly relies on the bioavailability and scavenging ability of the antioxidant machinery provided by cumulus cells [157]. Studies on the association between ART outcomes and oxidative stress-related biomarker through anti-oxidant measurement are summarized in Table 3.

**Table 3:** Studies on the association between ART outcomes and oxidative stress-related biomarker through anti-oxidant measurement.

Biomarker	Samples	N	Population (Inclusion criteria)	Exclusion criteria	Ovarian stimulation	Method	Positive findings	Negative findings	Reference
TAC/SOD/CAT/GR/GPx	FF	340	Endometriosis (n = 200), tubal (n = 140) 26-40 y/o BMI<25 kg/m^2^; infertility duration >2 years	N.A.	long protocol	CL (TAC) Spectrophotometer (SOD/CAT/GR/GPx)	N.A.	pregnancy	[112]
TAC/GSH/Vitamin C	FF	117	IVF	N.A.	long agonist (n = 38); short agonist (n = 4) antagonist (n = 36)	assay kits	fertilization rate (GSH in ICSI)	fertilization (cIVF), rates of good quality embryos, blastocysts, good quality blastocyst	[17]
GST	cGC	102	IVF	<23 or > 40 y/o, diabetes	antagonist	TBA	NOR	N.A.	[127]
TAC	FF	63	IVF	N.A.	long protocol	FRAP	Fertilization, embryo viability	oocyte retrieval	[158]
TAC	FF	41	male factor (n = 16), tubal factor (n = 17), idiopathic (n = 3), ovulatory factor (n = 5)	N.A.	long protocol	CL	pregnancy rate	oocyte maturity, fertilization, cleavage, embryo quality	[123]
TAC	FF	48	Tubal, 26-40 y/o partners/donors with normozoospermia and optimal ROS levels	contaminated with blood/media; more than one oocyte/no oocytes	long protocol	CL	oocyte quality, fertilization rate, and embryo quality	N.A.	[109]
TAC	FF	138	ICSI	N.A.	long protocol	colorimetric assay	pregnancy	oocytes retrieved	[111]
TAC	FF	100	PCOS (n = 43), tubal (n = 57)	endometriosis, male factor, unexplained infertility, diminished ovarian reserve	GnRH antagonist (PCOS); varying (tubal)	ELISA	N.A.	miscarriage, oocytes retrieved, fertilization, cleavage, grading of embryos	[115]

TAC: total antioxidant capacity; FRAP: ferric reducing ability of plasma; SOD: superoxide dismutase; CAT: catalase; GR: glutathione reductase; GPx: glutathione peroxidase family; GST: Glutathione-S-Transferase; GSH: glutathione; CL: chemiluminescence; FF: follicular fluid; cGC: cumulus granulosa cell; PCOS: polycystic ovarian syndrome; TBA: thiobarbituric acid; N.A.: not applicable.

#### 5.3.1 Non-enzymatic antioxidant capacity

Vitamins A, C and E, or endogenous compounds such as thiols, are all considered potent nonenzymatic antioxidants [19]. However, it is not easy to measure each non-enzymatic antioxidant component individually, and there are potential interactions among different antioxidant components in the biological fluids. Thus, total antioxidant capacity (TAC), also called nonenzymatic antioxidant capacity, has been widely used to indicate the potential of biological fluids to counteract the endogenously generated ROS effects [159]. It is defined as the moles of oxidants that are neutralized by 1 L of a test solution [159]. TAC allows the measurement of the net effect of all the antioxidants in the biological fluid instead of quantifying the activity of each agent individually [160,161].

Several studies have revealed correlations between decreased FF TAC and poor oocyte/embryo quality and low fertilization rate [109,158]. Some others showed no significant relevance between FF TAC and oocyte maturity, fertilization and pregnancy rate [112,115,123]. Thus, the association between TAC levels and IVF outcomes has been inconclusive. One common finding revealed by several studies shows no association between FF TAC and the number of oocytes retrieved [111,115,158]. These conflicting conclusions may result from different methods applied in different studies when determining TAC in FF, including chemiluminescence [109,123], ELISA [115], ferric reducing ability of plasma (FRAP) [158] and a colorimetric assay [111]. Although TAC measurement has advantages including simplicity, low cost and fast reactions [162], it has some drawbacks and is not recommended in clinical studies according to the recent published guideline [21]: First, The principle behind TAC measurement is based on the idea that the overall antioxidant capacity of a sample can be captured and expressed as a single numerical value, reflecting the combined contribution and interactions of all antioxidant components. Second, this method overlooks the fact that a significant portion of the body's antioxidant defense is provided by enzyme activities, which are not taken into account in this approach [163]. Therefore, the results of TAC only reflect the net effects coming from non-enzymatic antioxidants. Third, antioxidants and ROS are not directly correlated so they are weaker indicators of oxidative stress.

#### 5.3.2 Enzymatic anti-oxidants

To date, there is growing appreciation for the critical role of classical enzymatic anti-oxidants, such as SOD, CAT and GPx in folliculogenesis [164]. For instance, the level of GC catalase in dissected goat follicles of different stages was found to fluctuate concurrently in response to FSH during follicle development and mount in the large follicles. This suggests a role for catalase in dominant follicular selection [165]. A large body of literature aims to explore the association between levels of these classical enzymatic anti-oxidants and ART outcomes [112,166,167]. However, the results are so far inconclusive. For instance, the SOD activity in follicular fluid was higher compared to that in the serum, and a higher level of SOD activity was observed in patients whose oocytes did not become fertilized [166]. In contrast, Matos et al. demonstrated that ART success, determined by pregnancy and live birth, was associated with increased total SOD activity of cumulus GC [167]. Research conducted on 340 patients with endometriosis or tubal factors following long GnRH agonist reported no correlation between pregnancy outcome and follicular SOD and CAT levels [112]. These varying results can be explained by a lack of reference values for these antioxidants, thus making it impossible to determine whether the antioxidant levels are within the physiological ranges necessary for the normal oocyte and embryo development or not.

### 5.4 Newly emerging oxidative stress biomarkers and techniques

Recently, there are emerging molecular biomarkers that were found to be reflective of oxidative stress and relevant to IVF outcomes. By investigating patients undergoing modified natural cycle-IVF, Nagy et al. found HDL, the cardioprotective effects of which is believed to be partially attributed to its anti-oxidant properties, was also an important player in the FF anti-oxidative capacity. An increase in FF anti-oxidative function was associated with a reduced possibility of normal fertilization [168]. Peroxiredoxin 4 (Prdx4), which is traditionally viewed as an anti-oxidant enzyme to catalyze the reduction of ROS, was recently proposed as a potential biomarker to predict IVF outcomes. In a study on 138 women with tubal or male factor infertility undergoing their first IVF/ICSI cycle, follicular fluid Prdx4 protein level was indicative of higher fertilization rates, clinical pregnancy rates and live pregnancy rates [169].

Newly developed techniques, such as fluorescent nanodiamonds (FNDs) which have been traditionally used for applications such as labeling and drug delivery investigation, are also gaining more attention for being potential free radical biosensors [170]. By acting as magnetic sensors responding to the magnetic noise from free electron spins, FNDs are able to directly probe free radicals with spatial and temporal resolution [171]. With excellent biocompatibility and high sensitivity, FNDs have successfully been applied to perform localized free radical measurement in a variety of mammalian live cells [172,173], including boar sperm cells [174]. Thereby supporting the applicability of FNDs as biosensors in free radical research.

## 6 Methodological challenges in oxidative stress biomarker research in ART

Although numerous studies have investigated oxidative stress–related biomarkers in relation to ART outcomes, the findings remain heterogeneous and at times contradictory. Several methodological factors, already discussed throughout the literature, may at least partly explain these inconsistencies.

### 6.1 Influence of ovarian stimulation protocols

Most studies evaluating oxidative stress biomarkers have been conducted in women undergoing controlled ovarian hyperstimulation, including long and short GnRH agonist protocols or GnRH antagonist regimens. However, exogenous gonadotropin administration itself may alter systemic and follicular redox balance. Increases in serum paraoxonase 1 (PON1), SOD, GPx, and MDA activity have been reported following ovarian stimulation during IVF/ICSI cycles, irrespective of the stimulation strategy used [130,140]. Therefore, oxidative stress levels measured in hyperstimulated cycles may reflect not only intrinsic follicular physiology but also pharmacologically induced changes. Differences in stimulation protocol, gonadotropin dosage, duration of treatment, and number of retrieved oocytes may all influence the redox environment. As a result, comparisons across studies using different stimulation regimens should be interpreted cautiously.

In contrast, the modified natural cycle-IVF model yields a single dominant follicle and may better reflect physiological ovarian conditions. Using this model, Nagy et al. investigated follicular fluid composition and oocyte–embryo quality without the confounding effects of high-dose exogenous stimulation [168]. Such approaches may provide improved biological resolution when examining oxidative stress within a single follicular microenvironment.

### 6.2 Pooling of follicular samples and loss of follicle-specific resolution

In hyperstimulated cycles, follicular fluid from multiple follicles is commonly pooled per patient, and granulosa cells are often analyzed collectively. Consequently, oxidative stress biomarkers represent mean values across several follicles rather than measurements linked to a specific oocyte. Given that only approximately 16% of aspirated oocytes ultimately result in a live birth [104], substantial variability between follicles within the same patient is plausible. Pooling strategies obscure potential intra-patient heterogeneity and prevent direct correlation between a given follicular redox status and the developmental competence of its corresponding oocyte. Single-follicle models may therefore offer methodological advantages by preserving the biological association between follicular environment and oocyte outcome [168].

### 6.3 Variability in embryo culture conditions

Another important yet often overlooked factor relates to embryo culture conditions. Different IVF centers employ commercial embryo culture media whose composition is not fully disclosed and may vary considerably, particularly with respect to amino acids, pyruvate, and lactate concentrations [175].

Pyruvate and lactate serve as key substrates for embryo energy metabolism and are involved in maintaining reductive–oxidative balance during early development; disruption of these pathways has been shown to impair preimplantation embryo development in experimental models [176,177]. However, culture conditions are generally not incorporated into analyses exploring associations between oxidative stress biomarkers and ART outcomes. Variability in culture media composition may therefore contribute to inter-study differences and should be considered in future investigations.

### 6.4 Differences in biomarker selection and detection methodologies

Oxidative stress biomarkers encompass direct ROS measurements, oxidative damage products, and antioxidant capacity assessments. These approaches differ fundamentally in terms of the molecules detected, temporal resolution, and biological interpretation. Direct ROS detection methods, such as DCFH-DA fluorescence or chemiluminescence assays, are sensitive to experimental conditions, including reagent concentrations, temperature control, background luminescence, and injection techniques [[105], [106], [107], [108]]. Oxidative damage markers, including MDA, isoprostanes, 8OHdG, protein carbonyls, and AGEs, reflect downstream consequences of ROS exposure. However, widely used techniques such as TBA assays for MDA lack specificity [136,137], and commercial immunoassays for isoprostanes and 8OHdG may show variable performance and limited concordance with mass spectrometry–based quantification [21,134]. According to recently published guidelines, kit-based approaches should be interpreted cautiously, particularly when specificity and validation status are unclear [21]. Similarly, total antioxidant capacity (TAC) assays measure the net non-enzymatic antioxidant potential of biological fluids but do not account for enzymatic antioxidant systems and are not well standardized [21,163]. Consequently, TAC may not reliably represent the overall redox state and is not recommended as a standalone biomarker in clinical research [163].

### 6.5 Biological interpretation and infertility heterogeneity

Another layer of complexity arises from the dual role of ROS in ovarian physiology. A physiological level of ROS is indispensable for folliculogenesis, meiosis, and ovulation, whereas excessive ROS may be detrimental. Several studies suggest that moderate oxidative stress may be compatible with, or even associated with, favorable reproductive outcomes [131,139], highlighting the importance of distinguishing physiological from pathological redox states. Furthermore, associations between oxidative stress biomarkers and ART outcomes may differ according to infertility etiology. For example, follicular fluid isoprostanes were associated with miscarriage in PCOS patients but not in women with tubal factor infertility [115]. Such findings indicate that oxidative stress biomarkers may have context-dependent predictive value, and pooling heterogeneous populations without stratification may obscure clinically meaningful relationships.

## 7 Conclusion

Oxidative stress represents a biologically compelling yet methodologically complex domain in ART research. Evidence supports a dual role of ROS in ovarian physiology, where tightly regulated redox signaling is essential for folliculogenesis and ovulation, while excessive ROS impairs oocyte competence and embryo development. Importantly, ROS are not a uniform entity but comprise multiple species, including O_2_•^-^, H_2_O_2_, •OH, and reactive nitrogen species that differ in reactivity, lifetime, diffusion capacity, and biological targets. These species are generated through interconnected subcellular pathways, primarily originating from mitochondrial electron transport, NADPH oxidases, and enzymatic reactions, and can be further transformed via redox cascades. Their biological impact is therefore highly context-dependent, with certain species acting as signaling molecules, while others induce irreversible molecular damage. This complexity also implies that ROS dynamics may be selectively manipulated through targeted modulation of specific sources or antioxidant systems, offering potential therapeutic avenues but also introducing challenges in achieving precise control of redox balance.

Although numerous oxidative stress–related biomarkers have been proposed, none has yet achieved sufficient consistency, specificity, or standardization for routine clinical implementation. Current inconsistencies largely arise from heterogeneity in study design, stimulation protocols, biomarker selection, and analytical techniques. Moreover, the frequent pooling of follicular samples and lack of follicle-specific resolution obscure the biological relationship between redox status and individual oocyte outcome.

Future progress requires methodological harmonization, stratification by infertility etiology, and the development of technologies capable of directly probing ROS dynamics with spatial and temporal precision. Such advances may shift oxidative stress biomarkers from associative indicators toward mechanistically informative tools, ultimately improving prediction and modulation of oocyte competence in ART.

## CRediT authorship contribution statement

**Nuan Lin:** Conceptualization, Investigation, Methodology, Writing – original draft. **Koen van Zomeren:** Investigation, Writing – review & editing. **Torsten Plosch:** Conceptualization, Supervision, Writing – review & editing. **Naomi Hofsink:** Writing – review & editing. **Xiaoling Zhou:** Supervision, Writing – review & editing. **Uwe J.F. Tietge:** Conceptualization, Writing – review & editing. **Astrid Cantineau:** Conceptualization, Writing – review & editing. **Romana Schirhagl:** Conceptualization, Writing – review & editing. **Annemieke Hoek:** Conceptualization, Writing – review & editing.

## Declaration of competing interest

Unrelated to the current work, prof. A. Hoek, is member of an advisory board on the development and application of a lifestyle App for patients with infertility, Ferring Pharmaceutical Company, The Netherlands. R. Schirhagl is founder of QT Sense B.V. that commercializes quantum sensing equipment. This article has no direct relation to the work of QT Sense.

## Data Availability

No data was used for the research described in the article.
